# Autologous Cricoid Cartilage as a Graft for Airway Reconstruction in an Emergent Technique - A Case Report

**Published:** 2016-03

**Authors:** Farzad Izadi, Reza Vaghardoost, Vita Derakhshandeh, Behnam Sobouti, Yaser Ghavami

**Affiliations:** 1 *Department of Otorhinolaryngology, Iran University of Medical Sciences, Tehran, Iran.*; 2 *Department ofAesthetic and Reconstructive, Iran University of Medical Sciences, Tehran, Iran.*; 3 *Burn Research Center, Shahid Motahari Hospital, Iran University of Medical Sciences, Tehran, Iran*; 4 *General Practitioners, Research Assistant, Iran University of Medical Sciences and Health Services, Tehran, Iran.*

**Keywords:** Airway trauma, Autologus Cricoid Cartilage, Reconstruction

## Abstract

**Introduction:**

Laryngotracheal stenosis can be caused after traumatic injuries to the neck from the subglottic larynx to the trachea. Patients with laryngotracheal stenosis often need a tracheotomy and occasionally may become tracheotomy dependent. Different procedures have been described for the management of these lesions. Management options include techniques of endoscopic dilation, laser resection, laryngo-fissure, and an innovative array of plastic reconstructions with or without the use of stents.

**Case Report:**

This paper presents airway reconstruction in a young patient with severe subglottic stenosis due to a blunt trauma to the neck, who was treated using particles of an autologous fractured cricoid cartilage as the source for airway augmentation. An incision was made in the anterior midline of the cricoid lamina and deepened through the scar tissue to the posterior cricoid lamina. Then two lateral incisions (right & left) were made in the cricoid lamina and fractured cartilage particles and the scar tissue were removed via these two lateral incisions. The mucosal lining at the right and left of the midline incision, after debulking, were sutured to a lateral position. Thereafter three cartilage particles were used to reconstruct the anterior cricoid lamina and augment the lumen.

**Conclusion:**

It is worth to mention that an autologus cartilage graft can be used for certain cases with traumatic airway stenosis. Further follow up and more patients are needed to approve this method of reconstructive surgery in emergent situations.

## Introduction

External laryngeal trauma is a life threatening injury and if not promptly recognized and adequately treated, can also cause significant long term morbidity, owing to any combination of dysphonia, aspiration, and airway stenosis.

Successful repair of airway stenosis requires establishing an adequate airway while preserving the laryngeal functions of airway protection, phonation, and sustained glottic closure to increase intrathoracic pressure (1). Management options include techniques of endoscopic dilation, laser resection, laryngofissure, and an innovative array of plastic reconstructions with or without stenting (2).This paper presents a new method of cricoid reconstruction using the patient’s own cricoid cartilage remnants.

## Case Report

**Fig.1:**
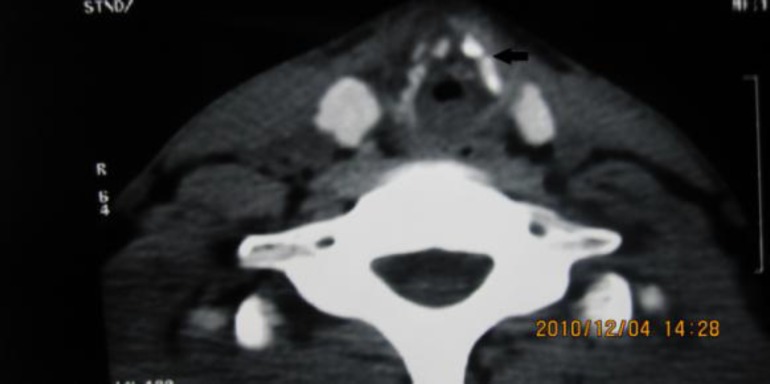
Subglottic airway, pre-op CT scan, showing a distorted airway. Fractured Cricoid cartilage (arrow

 A 21-year-old male experienced a blunt neck trauma due to a horizontal impact (clothes line injury). Twenty days after trauma, he developed respiratory distress and an urgent tracheotomy was performed for him in an emergency center. Then he was referred to our center for complementary and definitive treatment. One month after treatment, evaluation by direct laryngoscopic examination under general anesthesia revealed severe subglottic stenosis (grade 3 Cotton– Meyer) (Fig.1).

**Fig2:**
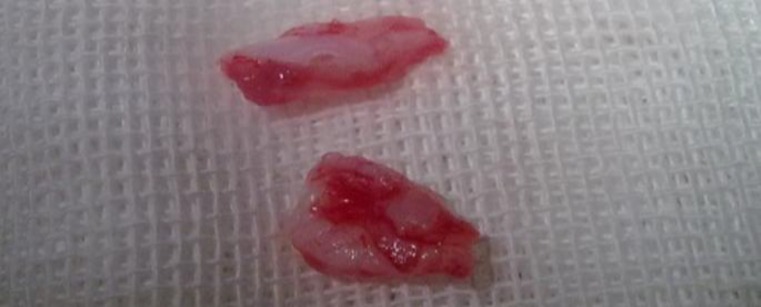
Cricoid cartilage particles

**Fig 3:**
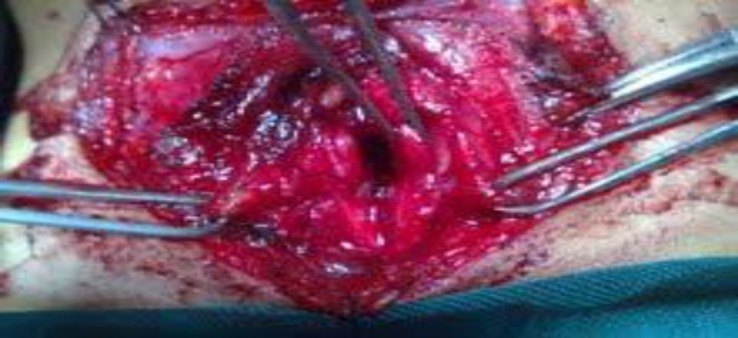
Extracting the fractured cartilage

**Fig 4:**
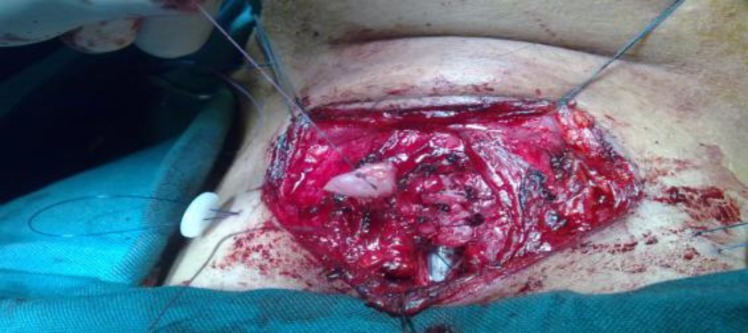
Stent is seen in the endolarynx, and the graft is to be placed in its final position

Stenosis dilation was executed at the same time, but decannulation could not be performed. Four months later he was scheduled for open laryngofissure intervention. During the laryngofissure procedure, severe narrowing of the subglottic airway was observed due to multiple cricoid fractures and scar tissue formation. After exposing the laryngeal skeleton, an incision was made in the anterior midline of the cricoid lamina and deepened through the scar tissue to the posterior cricoid lamina. Then two lateral incisions (right & left) were made in the cricoid lamina. While preserving the mucosa in the midline incision edges bilaterally, the fractured cartilage particles and the scar tissue were removed via these two lateral incisions. The mucosal linings on the right and left side of the midline incision were sutured to a lateral position after debulking. Thereafter three cartilage particles were used to reconstruct the anterior cricoid lamina and to augment the lumen (Figs 2,3,4). Before arranging the cartilage particles, a vertical limb of a Montgomery T- tube was inserted as a stent in the reconstructed subglottic area and secured on both sides of the neck with two buttons. 

**Fig 5:**
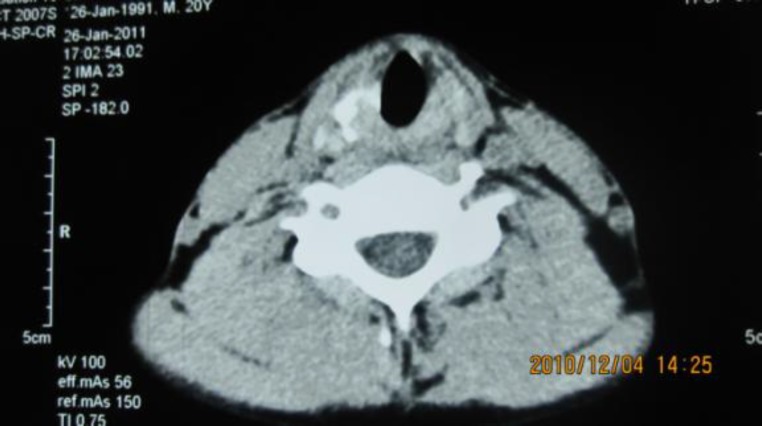
Post reconstructive subglottic airway

Forty days later, during an endoscopic approach, the stent was removed, some granulation tissue was ablated by laser and mitomycin-C was applied to the site of operation. Again two months later, during a second look through endoscopy, mild granulation formation was treated with CO₂ laser and mitomycin-C and the tracheotomy site was sealed. Since the patient underwent this last operation, 6 months ago, he has endured mild respiratory difficulty and some breathy voice due to an untreated vocal cord paralysis. Currently the patient has a good quality of life (Fig. 5).

In summary, our young patient developed near total subglottic obstruction after a blunt trauma to the neck (clothes-line injury). Conservative procedures could not resolve his problem, so we carried out an open laryngeal surgery and augmentation of the stenotic airway with a cartilage graft.

During the operation, some deformed fragments of cricoid cartilage that caused airway narrowing were removed, reshaped, and used as the source for a graft to augment the stenotic airway. About 6 months after injury, airway examination revealed an adequate lumen in the subglottic area, which provided a good quality of life for the patient. 

## Discussion

The most common cause of laryngotracheal stenosis is external trauma to the neck and prolonged endotracheal intubation. Both may result in acute and chronic stenosis, which occurs through different patho-physiological processes. When the laryngotracheal complex is injured by external trauma, disruption of the cartilaginous framework usually occurs as a consequence. The goal of any surgical procedure addressing laryngotracheal stenosis is to establish a satisfactory airway and eventually to decanulate the patient (3). 

Management options vary according to the timing, location, and severity of the stenosis, and include endoscopic management, mostly with the use of CO₂ laser and application of mitomycin-C, or dilation, that are used for mild to moderate cases and provide the best result with low morbidity. Open surgery is reserved for severe degrees of stenosis and is usually used after the conservative methods have failed (4). 

It is important to consider whether the site of stenosis requires reestablishment of the structural support usually by repositioning the existing cartilage or more commonly through the use of other cartilages or bone grafts. One of the most applied surgical techniques is resection and anastomosis of the stenotic segment, as a single stage operation of the narrowed airway. However, recurrence of the stenosis may develop, especially in the subglottic area. The cause of increased likelihood of restenosis in the subglottic area, rather than the trachea is not clearly known yet, but it may be due to rigid anatomy of the subglottic that may be more susceptible to inflammation and fibrosis compared to the trachea (5). 

In other literatures, graft cartilages were obtained from other resources such as ribs and costal cartilages for internal stenting (6-8); however, there is not any report for using the cricoid cartilage for an autologous graft. In our advent procedure, we used the patient’s own cricoid cartilage to expand the subglottic area with 90% stenosis and eventually we were able to successfully decannulate the airway, approximately 100 days later. 

## Conclusion

Autologus cartilage graft can be used for certain cases with traumatic airway stenosis with special conditions. Further follow up and more patients are needed to approve this method of reconstructive surgery in emergent situations. 
